# The Vascular Effects of Sodium Tanshinone IIA Sulphonate in Rodent and Human Pregnancy

**DOI:** 10.1371/journal.pone.0121897

**Published:** 2015-03-26

**Authors:** Jude S. Morton, Irene J. Andersson, Po-Yin Cheung, Philip Baker, Sandra T. Davidge

**Affiliations:** 1 Department of Obstetrics and Gynaecology, University of Alberta, Edmonton, AB, Canada; 2 Women and Children's Health Research Institute and the Cardiovascular Research Centre, Edmonton, AB, Canada; 3 Department of Medicine, University of Alberta, Edmonton, AB, Canada; 4 Department of Physiology, University of Alberta, Edmonton, AB, Canada; 5 Department of Pediatrics, University of Alberta, Edmonton, AB, Canada; 6 Gravida, National Research Centre for Growth and Development, Liggins Institute, University of Auckland, Auckland, New Zealand; University of Southampton, UNITED KINGDOM

## Abstract

Danshen, in particular its derivative tanshinone IIA (TS), is a promising compound in the treatment of cardiovascular diseases and has been used for many years in traditional Chinese medicine. Although many actions of TS have been researched, its vasodilator effects in pregnancy remain unknown. There have been a few studies that have shown the ability of TS to reduce blood pressure in women with hypertensive pregnancies; however, there are no studies which have examined the vascular effects of TS in the pregnant state in either normal or complicated pregnancies. Our aim was to determine the vasoactive role of TS in multiple arteries during pregnancy including: rat resistance (mesenteric and uterine) and conduit (carotid) arteries. Further, we aimed to assess the ability of TS to improve uterine blood flow in a rodent model of intrauterine growth restriction. Wire myography was used to assess vascular responses to the water-soluble derivative, sodium tanshinone IIA sulphonate (STS) or to the endothelium-dependent vasodilator, methylcholine. At mid-pregnancy, STS caused direct vasodilation of rat resistance (pEC_50_ mesenteric: 4.47±0.05 and uterine: 3.65±0.10) but not conduit (carotid) arteries. In late pregnancy, human myometrial arteries responded with a similar sensitivity to STS (pEC_50_ myometrial: 3.26±0.13). STS treatment for the last third of pregnancy in eNOS^-/-^ mice increased uterine artery responses to methylcholine (E_max_ eNOS^-/-^: 55.2±9.2% vs. eNOS^-/-^ treated: 75.7±8.9%, p<0.0001). The promising vascular effects, however, did not lead to improved uterine or umbilical blood flow *in vivo*, nor to improved fetal biometrics; body weight and crown-rump length. Further, STS treatment increased the uterine artery resistance index and decreased offspring body weight in control mice. Further research would be required to determine the safety and efficacy of use of STS in pregnancy.

## Introduction

*Salvia miltiorrhiza*, also known as Danshen, is a promising and increasingly researched traditional Chinese herbal medicine that has been widely used for many years to treat various disorders including coronary artery disease, cerebrovascular disease, myocardial infarction, angina pectoris, stroke and atherosclerosis [1]. Danshen has many components, including approximately 30 lipophilic diterpenes and >15 hydrophilic phenolic acid derivatives [2, 3]. Of the former category, tanshinone IIA (TS) is one of the most pharmacologically active and widely investigated. Studies have uncovered an impressive array of actions of TS including, but not limited to: vasodilation, inhibition of inflammatory mediators, oxidative stress and matrix metalloproteinases-2 and -9, as well as scavenging of peroxyl radicals and reduction of cardiac hypertrophy (reviewed in [4]). Due to its traditional cardiovascular uses, much of the vascular function work performed to date has been centered on the effects of TS on the coronary vasculature or the development of atherosclerosis. TS has been shown to cause vasodilation of mouse, rat and pig coronary arteries that was endothelium-, nitric oxide (NO)-, epoxyeicosatrienoic acid (EET)- or large-conductance, calcium-activated potassium channel (BK_Ca_)-dependent [5–9]. TS also reduced atherosclerotic plaques and lesions in the aortae of ApoE^-/-^ mice through a reduction of superoxide production, oxidized LDL, cholesterol and pro-inflammatory cytokines [10, 11]. In addition, researchers have shown TS-induced vasodilation of thoracic aortae from Sprague Dawley rats that was NO- and estrogen-dependent [12]. Although the effect of Danshen, a preparation of the whole root, has been studied in coronary [5–9] and femoral [13, 14] arteries, the role of the isolated TS component, or its water-soluble derivative sodium tanshinone IIA sulphonate (STS), in vascular resistance has not been extensively investigated. Our own group has shown STS-induced vasodilation of mesenteric arteries from male Sprague Dawley rats that was partially mediated by small- and intermediate-conductance calcium activated potassium channels (SK_Ca_ and IK_Ca_) but had no reliance on the NO pathway [15]. The precise mechanisms of (S)TS-induced vasodilation are, therefore, currently not fully elucidated.

To date, there have been three clinical studies which showed positive maternal effects of TS on hypertension in pregnancy [16–18]. In these studies, TS treated subjects demonstrated a reduction in morbidity, mean arterial pressure and blood viscosity, cholesterol and lipoprotein. There are no studies, however, which have examined the vascular effects of TS in the pregnant state in either normal or complicated pregnancies, particularly with regard to intrauterine growth restriction (IUGR). IUGR represents a failure of the fetus to reach its genetically determined potential size. IUGR affects up to 10% of all pregnancies worldwide [19, 20] and currently has no treatment. IUGR fetuses and the surviving infants have lifelong health issues including, among others, cardiovascular and metabolic complications (reviewed in [21, 22]). IUGR can occur when the transport mechanisms of nutrients and oxygen to the fetus are compromised. There are several up-stream causes of impaired nutrient transfer to the fetus including maternal, fetal and placental factors (reviewed in [23, 24]). Adaptations of the maternal cardiovascular system, such as an increase of uterine artery blood flow, allow for increased transfer of nutrients across the placenta, however, an increased uterine artery resistance has been associated with IUGR [25].

In light of its effects on oxidative stress, inflammatory state and vasodilation, (S)TS may provide a potential treatment strategy for compromised pregnancies which involve pathological changes in these areas. We hypothesized that STS would cause vasodilation of maternal resistance arteries, including the mesenteric and uterine vasculature, in pregnancy. Further, we hypothesized that treatment with STS would improve uterine artery vasodilation in an animal model of complicated pregnancy; the eNOS^-/-^ mouse model of intrauterine growth restriction, via actions on non-NO mediated vasodilator pathways.

## Methods

### Ethical approval

All protocols were approved by the University of Alberta Health Sciences Animal Policy and Welfare Committee in accordance with the guidelines of the Canadian Council on Animal Care and the Guide for the Care and Use of Laboratory Animals published by the US National Institutes of Health.

### I. Direct effects of STS—rodent studies

Three month old female Sprague-Dawley rats (Charles River, Wilmington, MA) were maintained on *ad libitum* standard rodent chow and tap water in a 10:14 hour light:dark cycle. Females were acclimatized in-house before breeding. Gestational day (GD) 0 was determined by the presence of sperm in a vaginal smear following an overnight introduction of a male. Rats (total n = 17) were euthanized by exsanguination via excision of the superior vena cava under inhaled isoflurane anaesthesia on GD 10.9±0.2 (term 22 days); corresponding to mid-gestation—an important clinical intervention time-point. Tissue dissections were performed in ice-cold physiological saline solution (PSS), composition (in mmol/l): 10 HEPES, 5.5 glucose, 1.56 CaCl_2_, 4.7 KCl, 142 NaCl, 1.17 MgSO_4_, 1.18 KH_2_PO_4_, pH 7.5. Small mesenteric (n = 64 artery sections from n = 14 animals) and main uterine branch (n = 56 artery sections from n = 13 animals) arteries were isolated for experimental procedures. The common carotid artery (n = 16 artery sections from n = 8 animals) was also assessed as a reference conduit artery to ascertain vascular bed specificity.

Arteries were cleaned of all surrounding adipose and connective tissues and 2 mm sections were mounted on two 40 μm wires attached to a wire myograph (DMT, Copenhagen, SV, Denmark) to allow isometric tension recordings. Vessels were normalized through a series of stepwise increases in diameter to determine their optimal resting tension, set to 0.8 x IC_100_ (the internal circumference equivalent to a transmural pressure of 100 mmHg).

Following a 30-minute equilibration period, vessels were twice exposed to a single dose of phenylephrine (PE, 10 μmol/l) followed by a single dose of methylcholine (MCh, 3 μmol/l) to check functional endothelial and smooth muscle integrity. A cumulative concentration-response curve to the adrenergic agonist phenylephrine (PE, 0.1 to 30 μmol/l, mesenteric and uterine arteries) or the thromboxane mimetic U46619 (U19, 0.01 to 3 μmol/l, carotid arteries) was performed. The EC_80_ (the effective concentration producing 80% of the maximum response) for each vasoconstrictor/artery combination was then determined.

Vascular responses to STS (1 to 100 μmol/l) were investigated following preconstriction with the EC_80_ concentration of the relevant vasoconstrictor. STS responses were performed in the absence or presence of inhibitors to investigate some of the possible vasodilator mechanisms. The three main endothelium-dependent vasodilator pathways; nitric oxide, prostaglandins and endothelial derived hyperpolarisation (EDH), were investigated using inhibitors of nitric oxide synthase (NOS) (*N*-nitro-L-arginine methyl ester hydrochloride, L-NAME, 100 μmol/l), cyclooxygenase (meclofenamate, 1 μmol/l), or a combination of apamin (100 nmol/l) and TRAM-34 (10 μmol/l) to block SK_Ca_ and IK_Ca_ channels, respectively. The inhibitors used are well-established and doses were chosen based on published methods [26–29].

In a separate series of experiments, the endothelium of mesenteric and uterine arteries was denuded using a knotted, human hair. The lack of endothelium was confirmed by assessment of responses to MCh (3 μmol/l). Vascular responses to STS (1 to 100 μmol/l) were then assessed following preconstriction with the EC_80_ concentration of the relevant vasoconstrictor.

### II. Direct effects of STS—human studies

Proof of principle studies were performed in a small number of human arteries (n = 12 artery sections from n = 3 patients) to determine if STS has vasodilator effects in human tissues from a relevant vascular bed. All human tissues were obtained from patients attending the Royal Alexander Hospital. All patients provided informed written consent to participate and the procedure was approved following full ethics review by the Alberta Health Services Ethics Committee, Edmonton. Signed consent forms were retained as documentation of participant consent. A myometrial biopsy from the uterus was obtained during scheduled caesarean sections of healthy pregnancies, mean gestational age 38 weeks and 6 days (st.dev. 4 days), without labour. The biopsy was immediately placed into ice-cold modified Kreb’s solution for collection and transportation. The tissue arrived in the laboratory and myometrial vessels were isolated within 60 minutes of delivery. Isolation of vessels and subsequent experimental protocols were performed in PSS as per animal vessels. Responses to the thromboxane mimetic U46619 (U19, 0.01 to 3 μmol/l) were investigated to assess the EC_80_ following which vascular responses to STS (1 to 100 μmol/l) were determined.

### III. In vivo administration of STS in mice

eNOS^-/-^ (strain B6.129P2-*Nos3* ^*tm1Unc*^/J) mice were chosen as an established model of IUGR that has been previously used in our laboratory. These mice have been shown to have increased blood pressure, decreased uterine artery blood flow and growth restricted offspring, both by ourselves and others [30–33]. C57BL/6J (control, n = 13) and eNOS^-/-^ (Jackson Laboratories, Bar Harbor, ME, n = 13) mice were maintained on *ad libitum* standard rodent chow and tap water in a 10:14 hour light:dark cycle. Females were acclimatized in-house before breeding. GD 0 of pregnancy was determined by the presence of a vaginal plug following an overnight introduction of a male. A subset of control (n = 8) and eNOS^-/-^ (n = 7) mice were treated with STS in their drinking water (approx. 27 mg/kg/day) from GD 12 to 18 (term 19 days). The dosage of STS was chosen following a literature review which determined treatment regimens of 80 mg/day in human (approx. 1.1 mg/kg/day) [34, 35] and ovine (approx. 1.8 mg/kg/day) [36, 37] trials and a median of 20 mg/kg/day in rodent studies [5, 10, 38–41]. The timing of treatment was chosen as a clinically relevant time-point during which intervention would be possible in humans.

Systolic blood pressure was measured by a validated tail-cuff system (IITC Life Science, CA, USA) on GD 17. Uterine and umbilical artery blood flow velocities were assessed *in vivo* in control and eNOS^-/-^ mice under anaesthesia on GD 17 using an ultrasound biomicroscope (model Vevo 2100, VisualSonics®, ON, Canada) as previously described [42, 43]. Briefly, uterine artery Doppler waveforms were obtained from both the left and right uterine arteries, and from the umbilical arteries of at least two fetuses. Peak systolic velocity (PSV) and end diastolic velocity (EDV) were measured from at least three cardiac cycles and the results were averaged. The uterine and umbilical vascular resistance indices were calculated from PSV and EDV measures.

On GD 18, mice were euthanized by exsanguination via cardiac puncture under inhaled isoflurane anaesthesia and the pregnancy outcome was determined by weighing and measuring the pups. The main uterine branch arteries were dissected in ice-cold PSS and prepared for experimental procedures on a wire myograph as described for studies in rat vessels. Following a 30-minute equilibration period, vessels were exposed to PE (10 μmol/l) and MCh (3 μmol/l) to check functional endothelial and smooth muscle integrity. Responses to the adrenergic agonist PE (0.0001 to 10 μmol/l) were performed to determine the EC_80_. Following constriction with PE, responses to the endothelium-dependent vasodilator MCh (0.0001 to 10 μmol/l) or the nitric oxide donor, sodium nitroprusside (SNP, 0.0001 to 10 μmol/l) in the absence or presence of L-NAME (100 μmol/l) were investigated.

### Statistical Analysis

All vascular function data were presented as mean ± standard error of the pEC_50_ (negative log of the effective concentration that will produce 50% of the maximum response) or the E_max_ (maximum response). Phenotypical parameters from ultrasound biomicroscopy, blood pressure measurements and offspring biometrics were presented as mean ± standard error. All data were normally distributed as assessed by the Kolmogorov-Smirnov test for Gaussian distribution. The significance of the difference in mean values of continuous variables between groups was determined by a one- or two-way analysis of variance (ANOVA), with Bonferroni post-test for multiple groups. A p value < 0.05 was considered statistically significant.

## Results

### Mechanisms of direct vascular responses to STS

**Fig 1:**
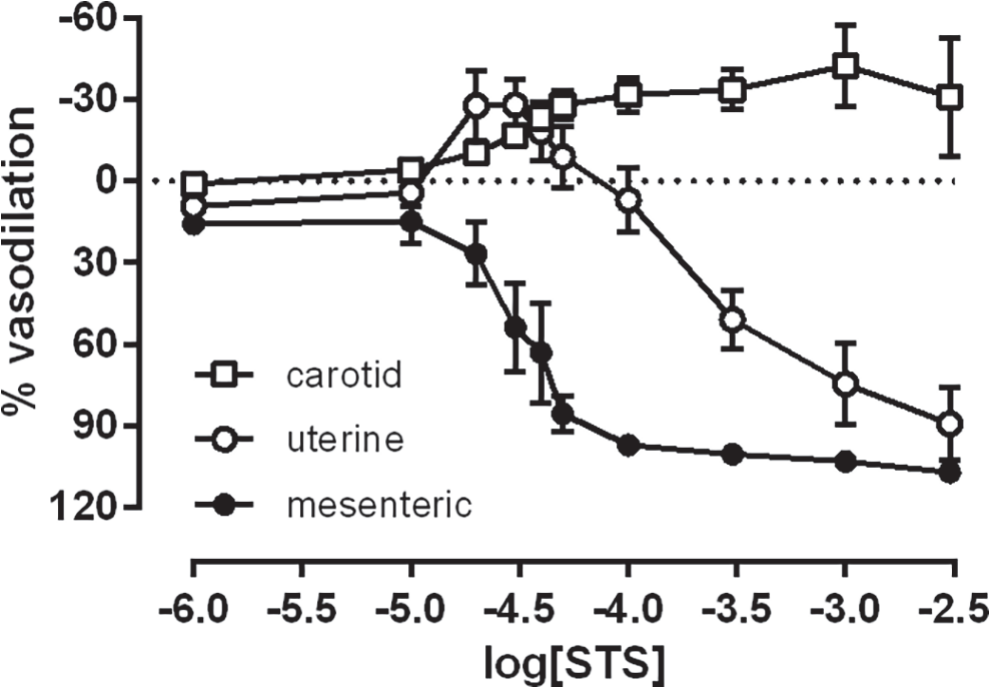
Direct vasodilator effects of STS in rat arteries. Vascular responses to STS in the carotid (open squares, n = 8), uterine (open circles, n = 10), and mesenteric arteries (closed circles, n = 8) from the pregnant rat (GD 10.9±0.2).

In mid-gestation rats, STS caused vasodilation of mesenteric resistance arteries (Fig. 1). Uterine arteries from mid-gestation rats demonstrated a biphasic response to STS. An initial contractile response (E_max_: -28.1 ± 9.4%) was followed by a vasodilator response that was significantly less sensitive than that in mesenteric arteries (p<0.0001, Fig. 1). Maximal vasodilation of mesenteric arteries and uterine arteries to STS were not significantly different. In the carotid arteries, STS caused only a vasoconstrictor response.

**Fig 2:**
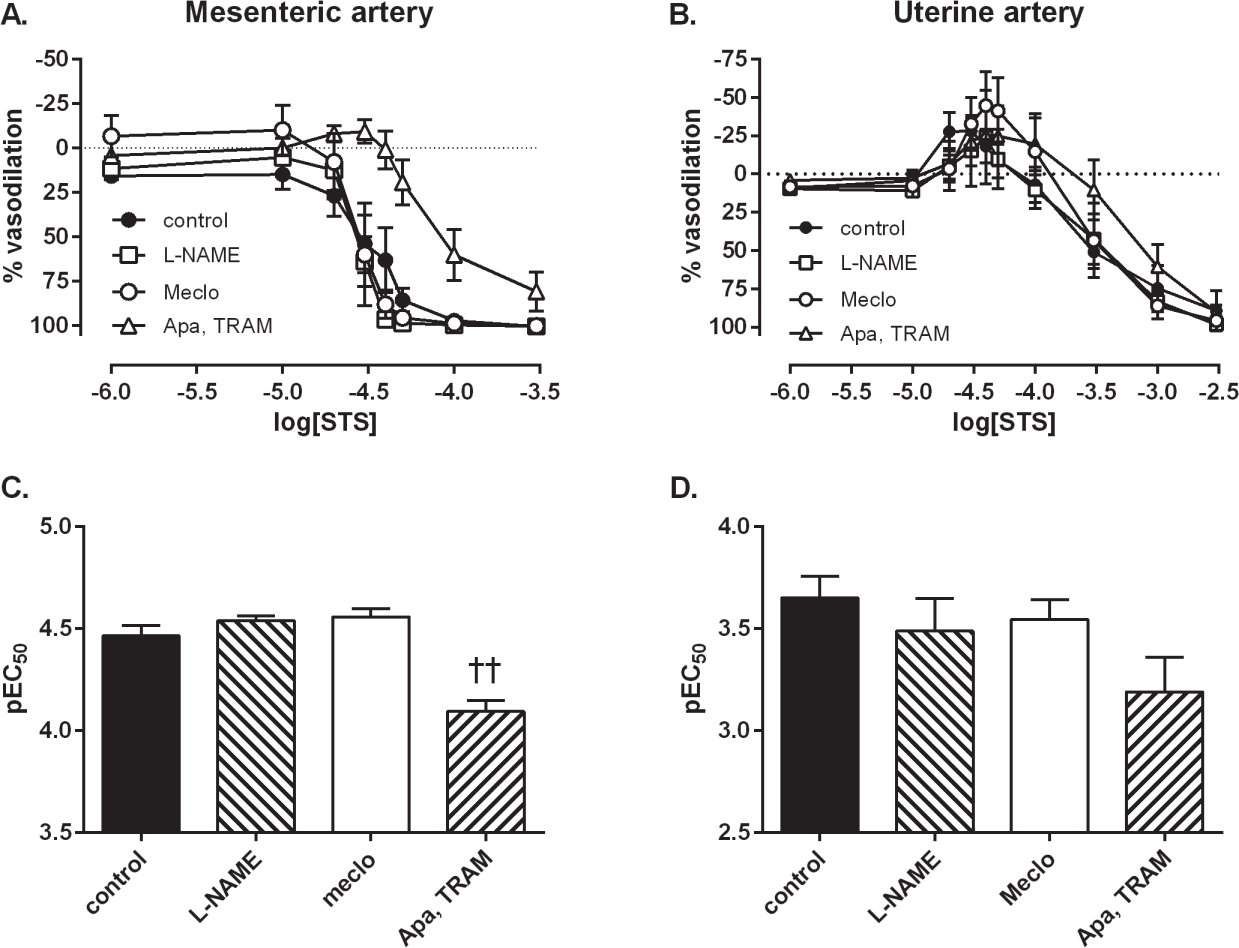
Involvement of endothelium-dependent vasodilator mechanisms in direct STS responses. Mechanisms of vascular responses to STS in mesenteric (A&C) and uterine (B&D) arteries from the pregnant rat (GD 10.9±0.2). Vasodilator responses to STS in the absence (closed circles) or presence of inhibitors of nitric oxide synthase (NOS) (*N*-nitro-L-arginine methyl ester hydrochloride, L-NAME, 100 μmol/l, open squares), cyclooxygenase (meclofenamate, 1 μmol/l, open circles) or a combination of apamin (100 nmol/l) and TRAM-34 (10 μmol/l) which block SK_Ca_ and IK_Ca_ channels respectively (open triangles) in A: mesenteric arteries and B: uterine arteries. Sensitivity (negative log of the effective concentration producing 50% of the maximal response) of vascular responses to STS in C: mesenteric arteries or D: uterine arteries. Data analysed by one-way ANOVA with a Bonferroni post-test; ††: p<0.01 vs. control vessel. Mesenteric: control n = 8; L-NAME n = 6; Apa, TRAM n = 7; Meclo n = 4. Uterine: control n = 10; L-NAME n = 6; Apa, TRAM n = 7; Meclo n = 5.

Inclusion of either the NOS inhibitor L-NAME or inhibition of COX using meclofenamate did not significantly affect STS-induced vasodilation of mesenteric arteries from mid-gestation rats. The presence of the potassium channel blockers apamin and TRAM-34, however, significantly inhibited STS-induced vasodilation compared to the control group (p<0.0001, Fig. 2A-B). In uterine arteries, inclusion of inhibitors for any of the three main endothelium-dependent pathways caused no significant change in STS-induced responses: including the biphasic vasoconstrictor and vasodilator components (Fig. 2C-D).

**Fig 3:**
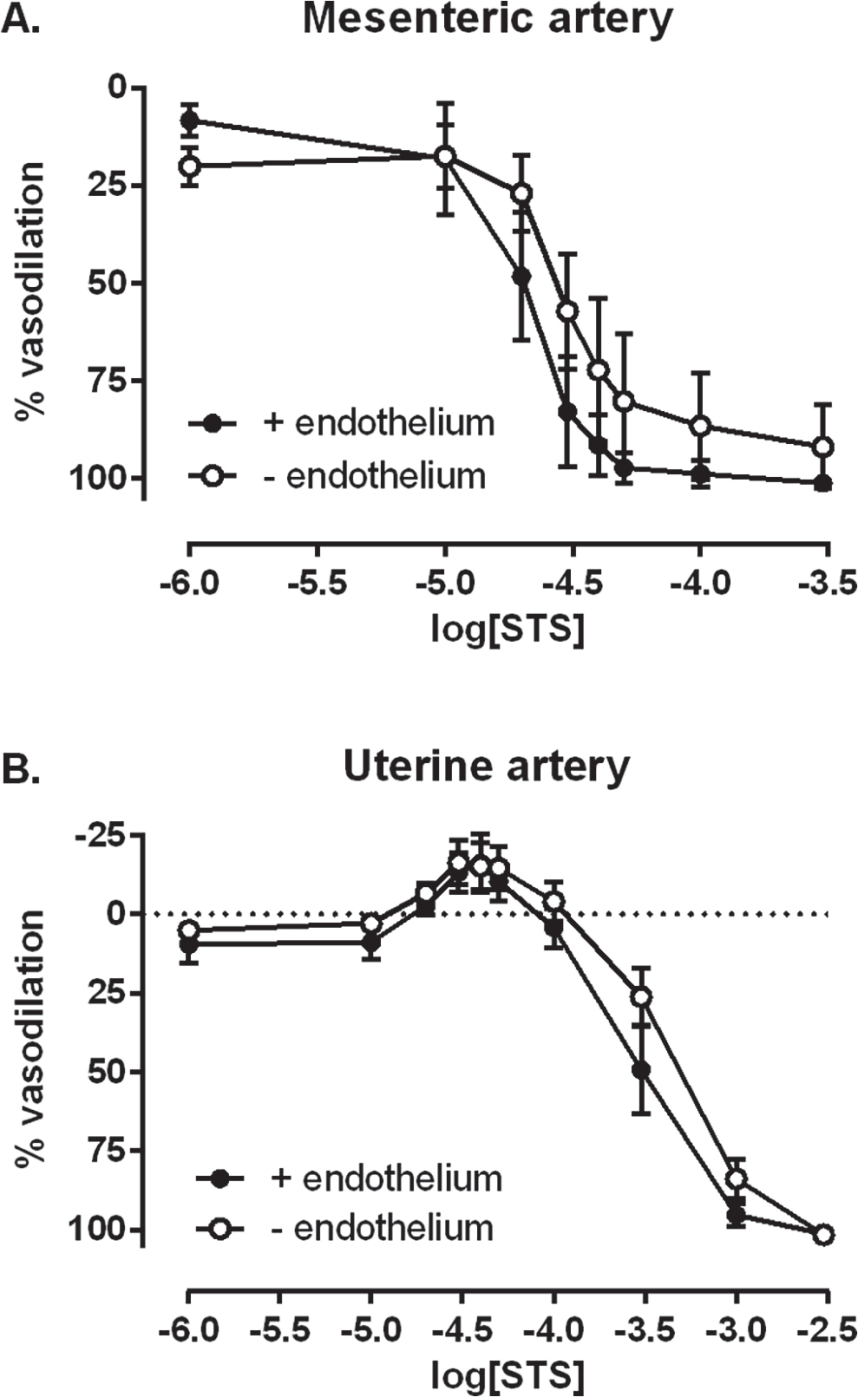
Endothelium-independent responses to STS. Vascular responses to STS in A: mesenteric arteries and B: uterine arteries from the pregnant rat (GD 10.9±0.2) with intact endothelium (closed circles) or following endothelial removal using a knotted human hair (open circles). Mesenteric intact n = 6; mesenteric denuded n = 5; uterine intact n = 4; uterine denuded n = 5.

Endothelium removal in mesenteric arteries resulted in a significant reduction in maximal vasodilator responses to MCh of 62.1 ± 7.4% (p<0.0001). Despite a 2.6-fold reduction in endothelial function, there was no significant change in sensitivity or maximal responses to STS following endothelium removal (Fig. 3A). Endothelium removal in uterine arteries also resulted in a significant reduction in maximal vasodilator responses to MCh of 80.0 ± 9.6% (p<0.0001). Despite a 5.7-fold reduction in endothelial function, there was no significant change in sensitivity or maximal responses to STS (Fig. 3B).

Human myometrial arteries responded to STS with a vasodilator response that was of similar potency (pEC_50_: 3.26 ± 0.13) and efficacy (E_max_: 89.07 ± 3.42%) to that observed in female pregnant rat uterine arteries. Human arteries, however, did not demonstrate the contractile component of the biphasic response observed in rat uterine arteries.

### Effect of *in vivo* STS treatment on vascular responses

**Fig 4:**
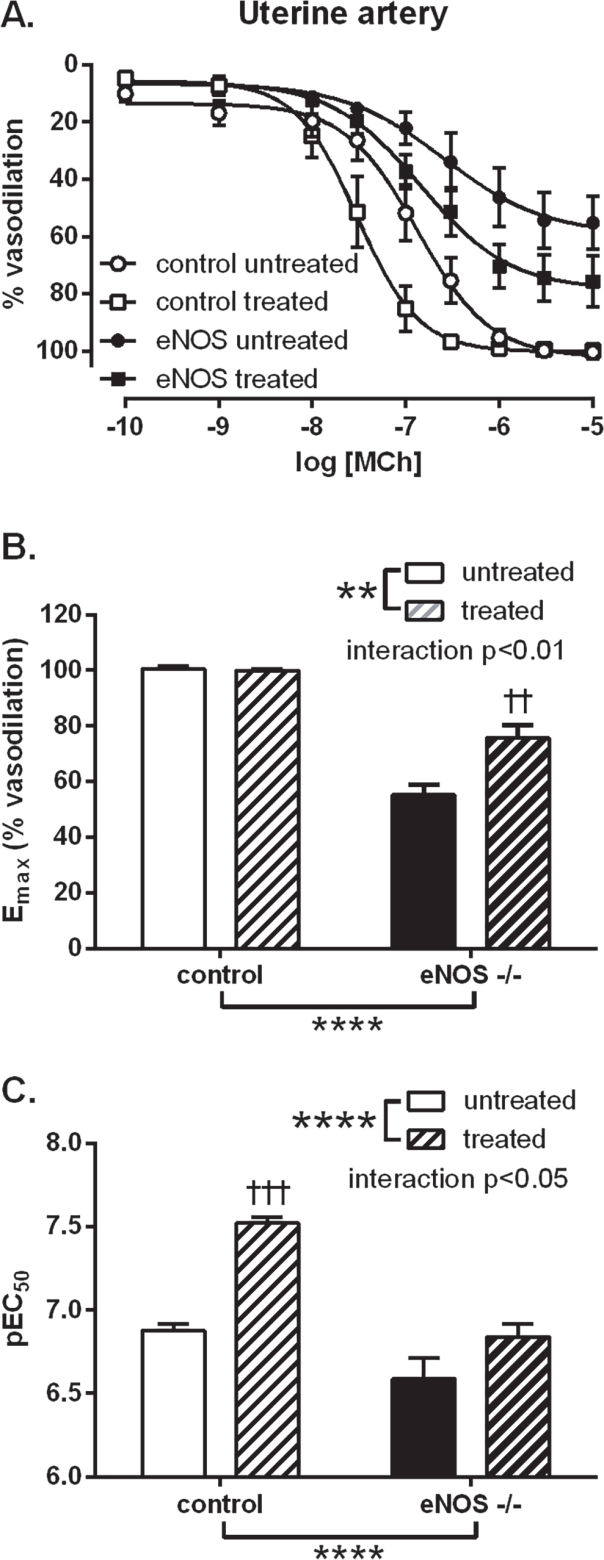
Effect of STS treatment on vasodilator responses in mouse arteries. A: Vascular responses of mouse uterine arteries to MCh from control (circles) and eNOS^-/-^ (squares) late pregnant (GD 18) mice, untreated (closed symbols) or treated (open symbols) with STS from GD 12 to 18. STS was given in drinking water at a dose of approx. 27 mg/kg/day. Summary data of B: maximal responses and C: sensitivity (negative log of the effective concentration producing 50% of the maximal response) to MCh. Data analysed by two-way ANOVA with a Bonferroni post-test; **: p<0.01 group treatment effect, ****: p<0.0001 group genotype effect, ††: p<0.01 vs. eNOS^-/-^ untreated, †††: p<0.001 vs. control untreated. Control untreated n = 5; control treated n = 5; eNOS^-/-^ untreated n = 6; eNOS^-/-^ treated n = 4.

Isolated mouse uterine arteries from GD 18 demonstrated reduced PE-induced vasoconstriction in eNOS^-/-^ mice that was increased following six days of STS treatment (E_max_ control untreated: 2.04 ± 0.31 mN/mm vs. treated: 2.94 ± 0.15 mN/mm; eNOS^-/-^ untreated 1.49 ± 0.13 mN/mm vs. treated 1.88 ± 0.19 mN/mm; group genotype effect p<0.001, group treatment effect p<0.01). MCh-induced vasodilation was also significantly reduced in eNOS^-/-^ mice compared to control mice (p<0.0001, Fig. 4A-B). Treatment with STS increased uterine arteries responses to MCh in both control (p<0.001) and eNOS^-/-^ mice (p<0.0001) (Fig. 4A-C). The increase in MCh-induced vasodilation was shown to be NO-independent using the NOS inhibitor, L-NAME (data not shown). In addition, SNP-induced vasodilation (pEC_50_ control: 7.36 ± 0.18 vs. eNOS^-/-^: 7.70 ± 0.13) was not altered in eNOS^-/-^ compared to control mice nor was it affected by STS treatment (pEC_50_ control treated: 7.50 ± 0.08; eNOS^-/-^ treated: 7.89 ± 0.11).

**Fig 5:**
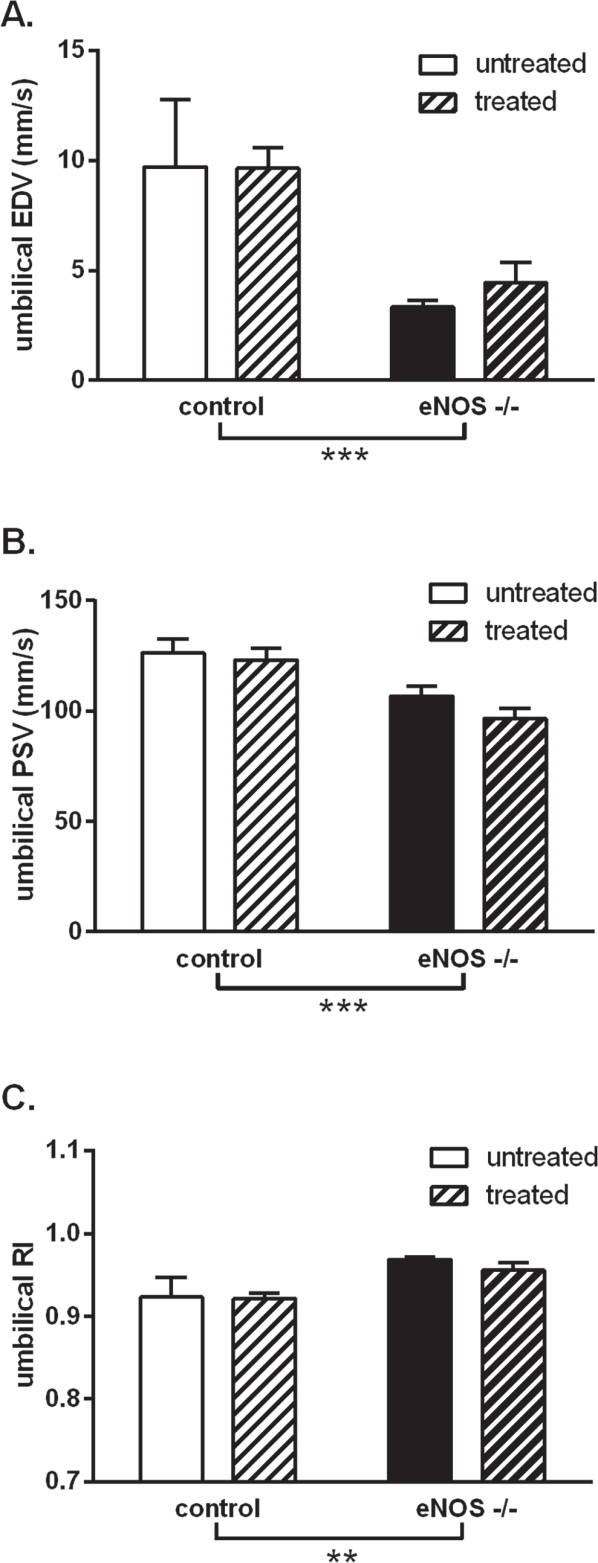
Effect of STS treatment on umbilical artery blood flow velocity in mice. Echocardiographic parameters of umbilical blood flow from control (white bars) and eNOS^-/-^ (black bars) late pregnant (GD 18) mice, untreated (open bars) or treated (hatched bars) with STS from GD 12 to 18. STS was given in drinking water at a concentration of approx. 27 mg/kg/day. A: end diastolic velocity (EDV), B: peak systolic velocity (PSV) and C: calculated resistance index (RI). Data analysed by two-way ANOVA with a Bonferroni post-test; **: p<0.01, ***: p<0.001, group genotype effect. Control untreated n = 4; control treated n = 8; eNOS^-/-^ untreated n = 6; eNOS^-/-^ treated n = 8.

Following the observation of direct STS-induced vasodilation and STS-mediated upregulation of MCh-induced vasodilation of the uterine vasculature, the ability of STS to improve uterine and umbilical artery blood flow and the outcomes of compromised pregnancy in an animal model of IUGR was tested. *In vivo*, the eNOS^-/-^ mice had a reduced umbilical artery end diastolic velocity (p<0.001, Fig. 5A) and peak systolic velocity (p<0.001, Fig. 5B), and a corresponding increase in the resistance index (p<0.01, Fig. 5C) for this vascular bed compared with control mice. Treatment with STS did not improve umbilical artery blood flow velocities in control or eNOS^-/-^ groups. In the uterine vasculature, these measures of blood flow were unaffected by the eNOS^-/-^ phenotype (peak diastolic velocity control: 525.2 ± 111.0 mm/s vs. eNOS^-/-^: 395.4 ± 28.5 mm/s; end diastolic velocity control: 283.8 ± 62.1 mm/s vs. eNOS^-/-^: 226.9 ± 20.3 mm/s). Contrary to our hypothesis, STS treatment led to a slight increase in the uterine artery resistance index (control untreated: 0.46 ± 0.02 vs. treated: 0.51 ± 0.02; eNOS^-/-^ control 0.43 ± 0.02 vs. treated 0.51 ± 0.04; group treatment effect p<0.05).

**Fig 6:**
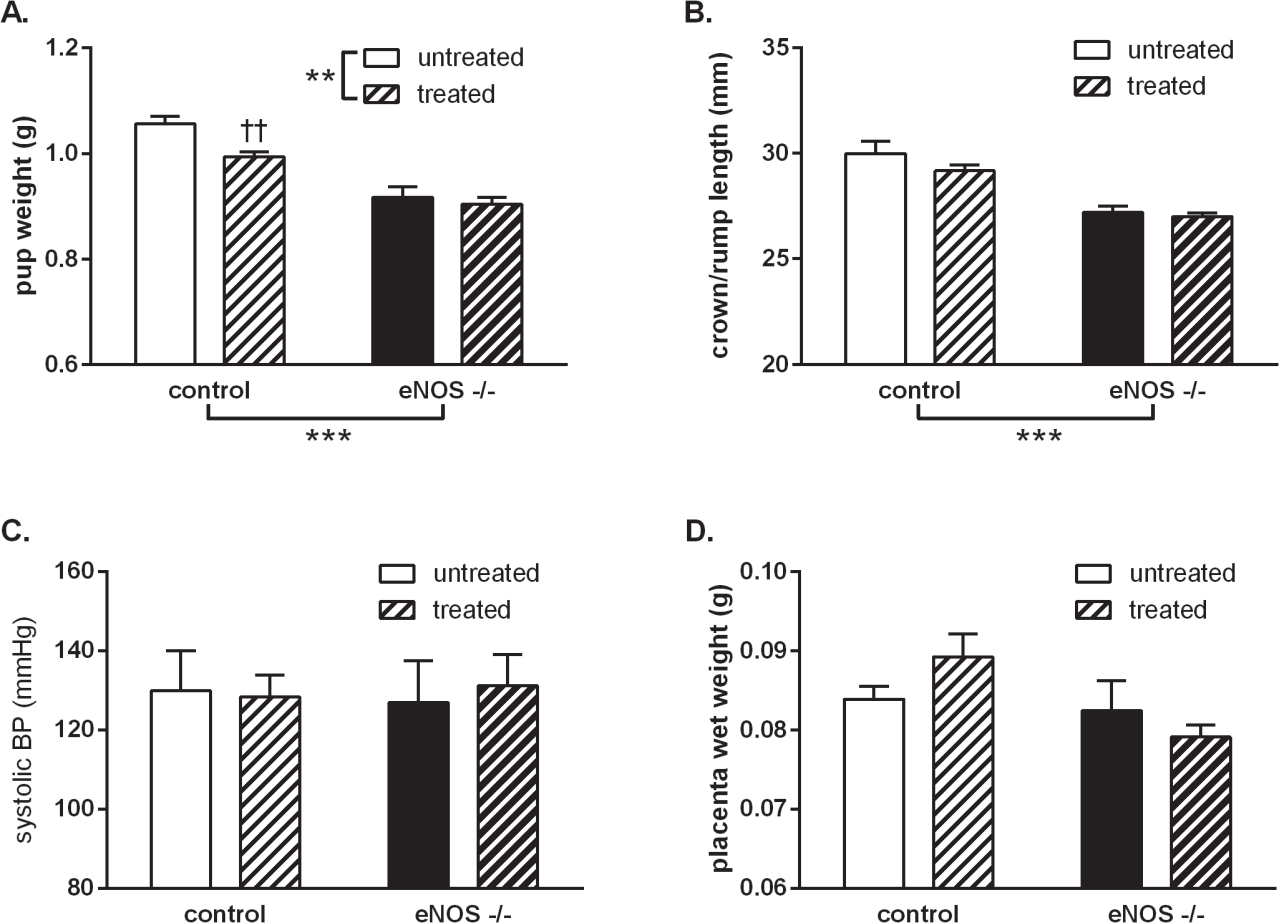
Effect of STS treatment on pregnancy outcomes in mice. Pregnancy outcomes in control (white bars) and eNOS^-/-^ (black bars) late pregnant (GD 18) mice, untreated (open bars) or treated (hatched bars) with STS from GD 12 to 18. STS was given in drinking water at a concentration of approx. 27 mg/kg/day. A: pup weight, B: pup crown to rump length, C: maternal systolic blood pressure and D: placental weight. Data analysed by two-way ANOVA with a Bonferroni post-test; **: p<0.01, ***: p<0.001, group genotype or treatment effects; ††: p<0.01 vs. control untreated. Control untreated n = 5(33); control treated n = 8(60); eNOS^-/-^ untreated n = 6(30); eNOS^-/-^ treated n = 7(43); where n = dams(pups).

In terms of pregnancy outcomes, the eNOS^-/-^ genotype caused a reduction in offspring birth weight (p<0.0001, Fig. 6A) and crown to rump length (p<0.0001, Fig. 6B) compared to control pregnancies. There was no change in maternal systolic blood pressure (Fig. 6C) or placental weight (Fig. 6D) with mouse genotype. Treatment with STS did not improve fetal growth in eNOS^-/-^ mice and, contrary to our hypothesis, STS treatment significantly reduced birth weight in control mice. STS treatment had no effect on maternal blood pressure or placental weight.

## Discussion

This study demonstrated a direct vasodilator effect of STS in vascular resistance arteries from pregnant rats. We have also demonstrated that STS was effective not only in dilating uterine arteries from the pregnant rat but also similarly dilates human myometrial arteries; providing proof of principle that STS may be effective in human pathology. Further, we showed that STS was able to improve the vasodilator capacity of uterine arteries when given *in vivo* to pregnant mice. The effect of STS on the vasculature, however, did not lead to improved uterine or umbilical blood flow *in vivo* or improved pregnancy outcomes.

STS caused non-NO dependent vasodilation of rat resistance (mesenteric and uterine) but not conduit (carotid) arteries. This was in line with our own studies in male rat mesenteric arteries [44] but in contrast to previous studies in the thoracic aorta and coronary circulations [8, 9, 12]; which demonstrated partially NO-mediated and endothelium-dependent vasodilation. In our study, STS-induced vasodilation in the mesenteric arteries was shown to be partly due to the small- and/or intermediate-conductance calcium-activated potassium channels (SK_Ca_ and IK_Ca_ respectively). Since the STS response was also shown to be endothelium-independent, these potassium channels were likely to be located on the smooth muscle. Interestingly, responses to STS in the mesenteric resistance vasculature from pregnant rats were consistent in efficacy, potency and sensitivity to apamin and TRAM-34 with our previous studies demonstrating vasodilator responses to STS in young male rats [44], suggesting that STS has sex-independent effects on the vasculature.

The effect of STS on the uterine vasculature during the pregnant state was of greater interest. Responses of rat uterine arteries to STS had a distinctly biphasic character that has also been observed in a previous study of rat pulmonary arteries [45]. In contrast to the mesenteric arteries, but in line with the pulmonary study, STS-induced responses were non-SK_Ca_ and—IK_Ca_, non-NO and non-prostanoid dependent; leaving their mechanism of action unknown. Consistent with the mesenteric arteries, responses in uterine arteries were also endothelium-independent. As anticipated following the observation of direct STS-induced vasodilation of rat uterine arteries, STS treatment was also shown to improve MCh-induced vasodilation of mouse uterine arteries. Improvement of vasodilation was observed in both the control and eNOS^-/-^ strains; providing additional support to the conclusion that STS-mediated effects on vasodilation are NO-independent.

The effect of STS was also assessed in the closest available human equivalent to the rodent uterine artery, the myometrial artery. Excitingly, not only were vasodilator responses observed, these responses in the myometrial artery were consistent in efficacy and potency to those observed in the rat uterine artery suggesting that the effects of STS were translatable from rat to human arteries.

A vasodilator of the uterine vasculature during pregnancy has potential clinical applicability, particularly for disorders that involve a decrease in placental and fetal perfusion. In particular, the endothelium-independence of STS responses provides a potential therapeutic approach in conditions characterized by endothelial dysfunction, such as preeclampsia or IUGR. Therefore, we next tested the ability of *in vivo* STS treatment to improve vascular function in a rodent model of IUGR. While there was increased uterine artery vasodilation following STS treatment of control and eNOS^-/-^ mice, we did not observe improved blood flow in the uterine or umbilical arteries during pregnancy nor did the vascular effects lead to improved fetal outcomes in either genotype. While the effect of *in vivo* STS treatment on isolated uterine artery function provides evidence that the treatment regimen was effective systemically, the multiple actions of STS and an integrated response of the tissues *in vivo* may have affected the outcome which was observed.

Several concerns regarding the use of STS in pregnancy were raised in regards to its effect on the control animals. STS treatment caused a biphasic response in rat uterine arteries which included almost 30% constriction prior to the vasodilator response. Further, adrenergic-mediated vasoconstriction in mouse uterine arteries was increased by STS treatment. These observations might suggest a risk for maternal hypertension or decreased uterine blood flow as a result of a hyper-constrictive phenotype. The increased adrenergic constriction following STS treatment, however, normalized the reduced PE constriction observed in untreated eNOS^-/-^ mice compared with untreated control mice. In addition, in our *in vivo* model, neither maternal systolic blood pressure nor uterine blood flow velocity were affected in either group of mice treated with STS. A previous study in pregnant sheep has demonstrated no maternal cardiovascular changes but a slight, significant increase in fetal systolic blood pressure in response to maternal treatment [46]; suggesting that the fetal effects of STS treatment need to be further explored. In our study, treatment with STS did cause a slight increase in the uterine resistance index in control and eNOS^-/-^ mice, however, the physiological significance of the magnitude of this change is questionable. Reassuringly, constriction to STS was not observed in human myometrial arteries and, therefore, might be a species specific effect. In our study, STS treatment caused a significant reduction in the body weight of control offspring, raising a further concern for its use during pregnancy. Further characterization of STS responses in human vessels and *in vivo* studies of the effect of STS in pregnancy and on the offspring would be necessary to determine the safety and efficacy of the *in vivo* use of STS during pregnancy.

In summary, we have demonstrated vasodilation to the Danshen derivative, STS, in resistance arteries that was consistent between rodent and human arteries. The *in vivo* effect of STS during healthy and complicated pregnancies, however, warrants further investigation. As discussed above, the non-NO, non-endothelium dependent mechanisms of STS-induced vasodilation observed in the current study may be of benefit in conditions which are known to be associated with endothelial dysfunction; such as preeclampsia and IUGR. Further research, however, is necessary to fully elucidate the effect of STS on uterine and feto-placental blood flow; particularly in regard to its effect on fetal outcomes. Given the current lack of treatments for pregnancy complications such as IUGR, the use of a novel compound such as STS that has been historically employed for other pathological conditions could provide a solution in an area of critical need.
